# Defining progression in erosive tooth wear: an interpretive challenge

**DOI:** 10.1007/s00784-026-07217-8

**Published:** 2026-10-13

**Authors:** Bryan D. Murchie, David Lynch, Andrew Carr, David Edwards

**Affiliations:** School of Dental Sciences, Faculty of Medical Sciences, Newcastle University, Framlington Place, Newcastle upon Tyne, NE2 4BW UK

**Keywords:** Erosive tooth wear, Intraoral scanner, Digital dentistry, Longitudinal studies, Decision making, Quantitative assessment

## Abstract

**Objectives:**

To critically evaluate current approaches to longitudinal erosive tooth wear (ETW) monitoring and examine whether quantitative intraoral scanner (IOS)-based measurements and conventional clinical indices can be meaningfully compared in defining progression.

**Materials and methods:**

A systematic review identified longitudinal studies evaluating ETW using IOS-based quantitative approaches alongside conventional clinical assessment methods. MEDLINE, Embase, Scopus, and Web of Science were searched from database inception to January 2026, with grey literature sources also searched. The electronic database searches were updated in August 2026 using the original search strategies. Reference-list screening and forward citation tracking were undertaken alongside both the initial search and the updated search.

**Results:**

Five longitudinal studies met the eligibility criteria. IOS-based approaches consistently detected measurable surface change over time, including during relatively short follow-up intervals in which conventional clinical indices frequently demonstrated no categorical progression. Substantial heterogeneity existed in progression thresholds, outcome measures, superimposition methodologies, and reporting strategies. Agreement between IOS-derived measurements and conventional index scores was variable and generally weak to moderate. IOS quantifies continuous dimensional change, whereas clinical indices categorise severity using ordinal scales. No validated framework was identified linking quantified dimensional surface loss to clinically meaningful ETW progression.

**Conclusions:**

Quantitative IOS measurements and conventional clinical indices assess different dimensions of tooth wear and cannot currently be interpreted as directly interchangeable measures of progression. Validated frameworks linking quantitative surface loss to biological and clinical relevance are required.

**Clinical relevance:**

IOS enables high-resolution longitudinal quantification of tooth surface change, but the clinical significance of detected change remains uncertain. Establishing clinically meaningful progression thresholds is essential for integrating quantitative digital monitoring into routine care.

**Supplementary Information:**

The online version contains supplementary material available at https://doi.org/10.1007/s00784-026-07217-8.

## Introduction

ETW is a multifactorial chemical-mechanical process characterised by cumulative, irreversible loss of dental hard tissue not caused by bacteria, with chemical erosion acting alone or in combination with mechanical factors including attrition and abrasion [1–3]. Physiological tooth wear represents a normal lifelong process, with reported mean annual rates estimated at 15 μm for premolars and 29 μm for molars [4]. In comparison, pathological wear is atypical for the patient’s age, which may result in functional impairment, aesthetic compromise, dentine hypersensitivity, reduced quality of life and, in advanced cases, complex restorative management [2, 4–6]. Global epidemiological evidence suggests that the prevalence and severity of tooth wear are increasing [3, 7–10], particularly among younger populations [4, 11, 12], raising concerns regarding early onset progression and long-term management.

Early identification and longitudinal monitoring of tooth wear are central to preventive care [6], especially as recent work has demonstrated measurable tooth loss following relatively short dietary acid exposure intervals [13, 14]. Conventional assessment predominantly relies on visual and tactile examination supported by clinical scoring systems, including the Basic Erosive Wear Examination (BEWE) and Smith and Knight Tooth Wear Index [6, 15–17]. Such indices were primarily developed to facilitate rapid clinical screening, severity categorisation, treatment decision-making, and longitudinal assessment within routine clinical and research settings [18–21]. However, by design, ordinal scoring systems compress continuous surface change into categorical classifications, potentially limiting sensitivity to small incremental changes occurring over shorter monitoring intervals [22, 23]. A further limitation of visual inspection is the resolution of the human eye [24]. Although some index-based systems incorporate treatment recommendations based on categorical severity, these thresholds remain largely consensus-driven and have not been directly validated against quantified longitudinal surface loss.

Advances in digital dentistry have introduced IOS-based approaches capable of generating high-resolution three-dimensional datasets for longitudinal surface comparison and quantitative wear analysis. These systems permit surface superimposition and quantitative measurement of linear or volumetric tissue loss over time [25, 26]. Emerging evidence suggests that IOS-based approaches may detect early surface changes that are not identified by conventional assessment [27, 28]. Digital monitoring may facilitate patient communication and support preventive interventions. Consequently, increasing interest has emerged regarding the potential role of IOS technologies in the longitudinal monitoring of ETW progression [29].

However, the increasing adoption of quantitative digital methodologies has also exposed important conceptual and clinical challenges. IOS-derived measurements quantify continuous dimensional surface change, whereas conventional clinical indices categorise severity using ordinal scales designed primarily for clinical interpretation and decision-making. As a result, these approaches assess fundamentally different dimensions of tooth wear progression despite frequently being interpreted as directly comparable measures of progression within the literature. Furthermore, substantial heterogeneity exists regarding progression thresholds, superimposition strategies, outcome measures, and the interpretation of clinically meaningful change.

**Fig. 1:**
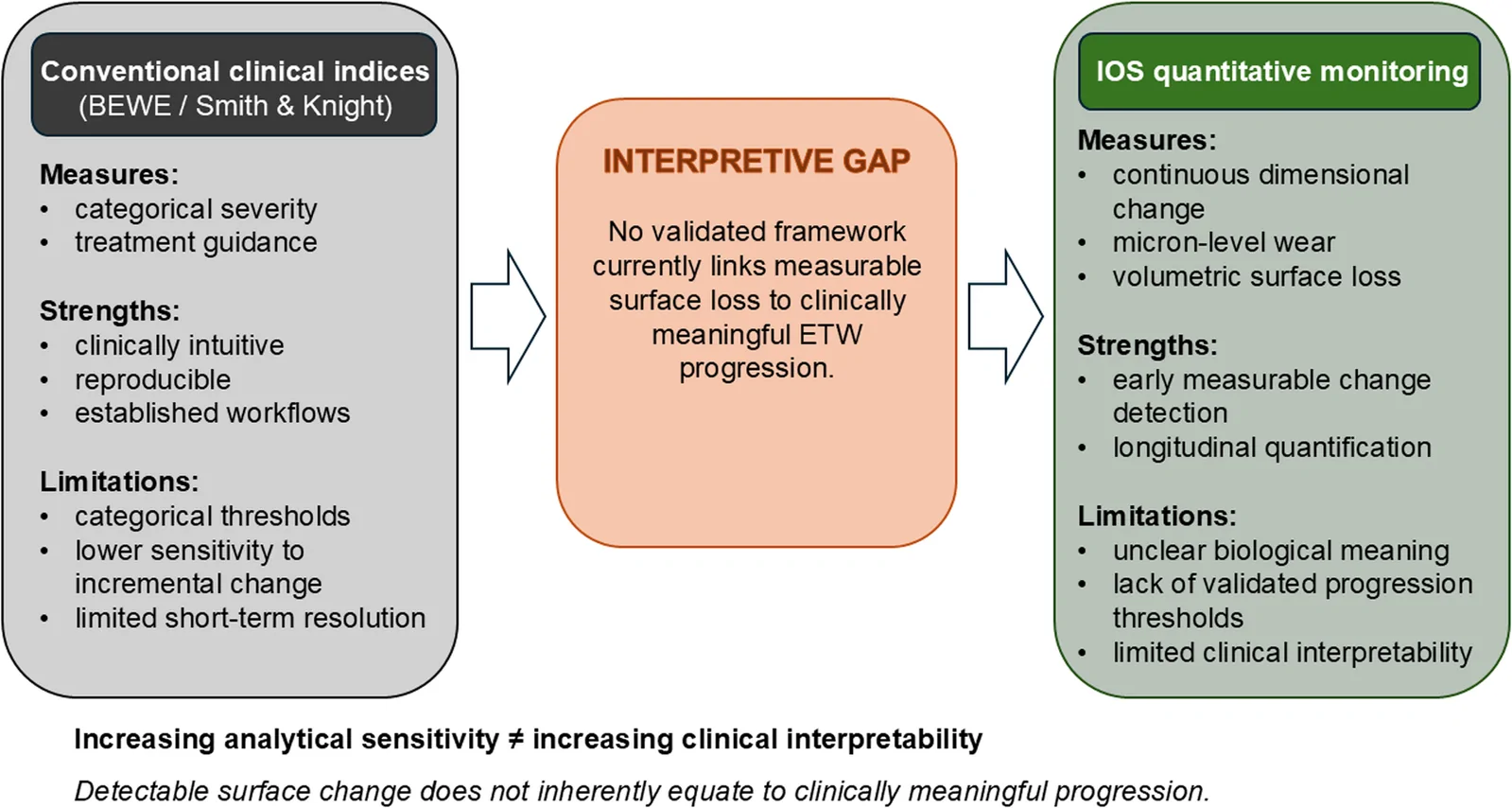
Conceptual framework illustrating the interpretive gap between quantitative digital monitoring and conventional clinical assessment of erosive tooth wear progression

Importantly, the detection of measurable surface change does not necessarily equate to clinically relevant progression. While IOS-based approaches may identify dimensional changes at micron-level resolution, the clinical and biological interpretation of such measurements remains poorly defined (Fig. 1). No validated framework currently exists linking quantified dimensional surface loss to biologically and clinically meaningful ETW progression.

Therefore, the aim of this systematic review was to evaluate current approaches to longitudinal ETW monitoring and critically examine how progression is defined, measured, and interpreted across quantitative digital and conventional clinical assessment methods.

## Review methodology

### Literature search

A systematic review was undertaken to identify longitudinal studies evaluating erosive tooth wear (ETW) using intraoral scanner (IOS)-based quantitative approaches alongside conventional clinical assessment methods. Electronic searches were conducted in MEDLINE, Embase, Scopus, and Web of Science from database inception to January 2026. Grey literature was additionally searched using OpenGrey, the Grey Literature Report, and ProQuest Dissertations & Theses Global. The electronic database searches were updated in August 2026 using the original search strategies to identify subsequently published studies. Reference-list screening and forward citation tracking were undertaken alongside both the initial search and the updated search to identify relevant studies not captured through the electronic search strategies. Full electronic search strategies are provided in Online Resource 1.

Search terms combined concepts relating to erosive tooth wear, intraoral scanning, digital monitoring, quantitative wear assessment, and conventional clinical indices, including BEWE and Smith and Knight methodologies [19, 30].

The systematic review was reported in accordance with PRISMA 2020 guidance. Given the substantial conceptual and methodological heterogeneity across the literature, synthesis was qualitative and critically interpretive rather than quantitative, and meta-analysis was not undertaken.

### Eligibility criteria

Studies were eligible for inclusion if they evaluated longitudinal ETW progression using IOS-based quantitative assessment alongside conventional clinical assessment approaches or index-based methodologies, and reported outcomes relating to progression detection, longitudinal monitoring, or interpretation of measured change. Studies evaluating IOS-based monitoring without comparison to conventional clinical assessment approaches were excluded, as the primary aim of the review was to critically examine how progression is comparatively defined and interpreted across quantitative and categorical monitoring frameworks. Both prospective and retrospective longitudinal clinical studies were eligible. In vitro longitudinal studies meeting the remaining eligibility criteria were also considered. Studies exclusively evaluating restorative materials, laboratory scanner accuracy, or non-longitudinal outcomes were excluded.

Studies employing indirect digital workflows involving digitisation of conventional impressions or casts were retained where IOS-derived digital surface analysis formed part of the longitudinal monitoring workflow. However, indirect acquisition pathways may introduce additional dimensional variability and should be interpreted cautiously when comparing quantitative progression outcomes.

### Data extraction and critical synthesis

The following variables were extracted where available: study design, participant characteristics, follow-up duration, IOS systems and digital workflows, conventional clinical assessment methods, progression thresholds, outcome measures, and approaches to interpretation of longitudinal change. Data extraction was performed independently by two reviewers, with discrepancies resolved through discussion and consensus.

Given the substantial heterogeneity in study methodology, progression definitions, and outcome reporting, formal quantitative synthesis was not undertaken. Accordingly, the review prioritised critical synthesis and interpretive analysis, with particular emphasis on differences in measurement construct, definitions of progression, clinically meaningful change, and methodological factors influencing longitudinal monitoring outcomes. The review did not undertake a formal evaluation of the measurement properties of individual instruments, as its primary objective was to examine the definition and interpretation of longitudinal progression across different monitoring approaches.

### Study selection

Two reviewers independently screened titles and abstracts against the predefined eligibility criteria. Potentially eligible articles were subsequently assessed independently at full-text level. Disagreements regarding study eligibility were resolved through discussion and consensus.

### Methodological quality assessment

Methodological quality of the included studies was appraised using the Newcastle-Ottawa Scale (NOS). Quality assessment was used to inform the critical synthesis, with emphasis placed on conceptual and methodological heterogeneity rather than comparative quality scores.

## Results

### Study selection

**Fig. 2:**
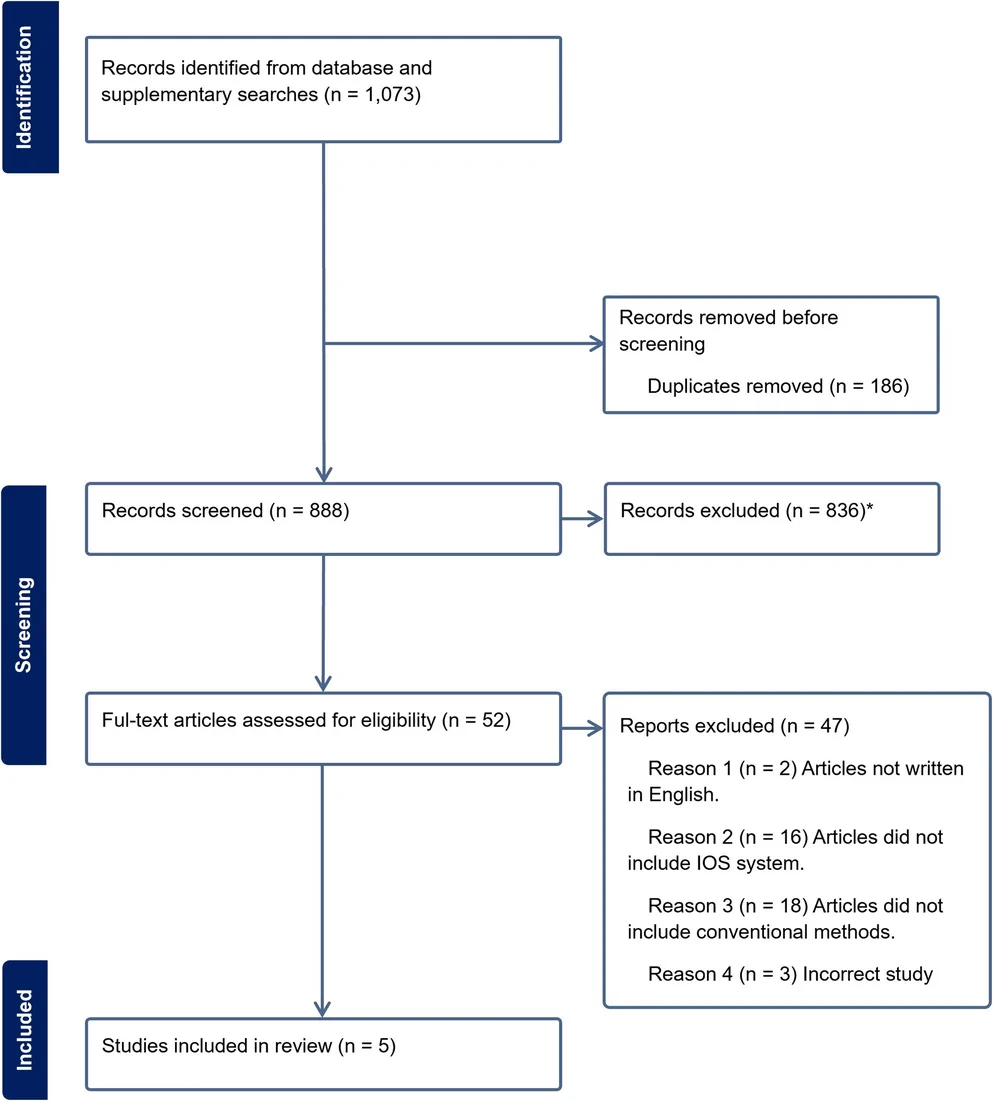
PRISMA flow diagram of study identification and screening

The study selection process is shown in Fig. 2. A total of 1,074 records were identified through electronic database and grey literature searching. Following removal of duplicates (*n* = 186), 888 records were screened by title and abstract, of which 836 were excluded. Fifty-two full-text articles were assessed for eligibility. Ultimately, five longitudinal studies met the inclusion criteria and were included in the qualitative synthesis. Although in vitro studies were eligible for inclusion, none met the final eligibility criteria. The August 2026 search update identified no additional studies meeting the eligibility criteria.

### Study characteristics

Key characteristics of the included longitudinal studies are summarised in Table 1, with additional methodological detail provided in Online Resource 1. Five longitudinal cohort studies (prospective or retrospective) were included [27, 28, 31–33]. Sample sizes ranged from 24 to 517 participants with follow-up periods of 1–25 years.

**Table 1:** Conceptual synthesis of longitudinal erosive tooth wear monitoring studies

Study	Digital workflow / surfaces	Follow-up	IOS findings	Conventional findings	Interpretation
Borș et al., 2025	MEDIT i700/Exocad DentalCAD (Galway 3.1), best-fit registration; maxillary anterior buccal and palatal surfaces	10 years	Quantified mean surface loss (~ 137 μm); greater palatal wear detected	BEWE identified progression but with limited resolution	Digital quantification detected incremental dimensional change beyond categorical BEWE progression
Ellingsgaard et al., 2025	TRIOS 3/TRIOS Patient Monitoring v21.1; tooth-by-tooth alignment; central incisors, canines and first molars	11–17 months (median 12 months)	Quantified measurable wear over a short interval	mBEWE showed no significant progression	Digital quantification demonstrated greater sensitivity to early short-term change
Marro et al., 2020	TRIOS-digitised casts/WearCompare; selective surface alignment; first-molar occlusal surfaces	24–35 months	Volumetric loss discriminated progression vs. non-progression surfaces	BEWE classified categorical progression	Quantitative digital analysis may support adjunctive assessment rather than replacement
Maluf et al., 2025	CEREC Omnicam-digitised serial casts/Geomagic Control; Best Fit alignment and 3D Compare; full-dentition occlusal wear	25 years	Large cumulative surface loss quantified longitudinally	BEWE detected progression with lower granularity	Quantitative and categorical methods demonstrated different progression behaviour
García et al., 2022	3M True Definition/Geomatic™; serial scan overlay/alignment and 3D colour-map comparison; buccal/vestibular, lingual/palatal and incisal/occlusal surfaces	12 months	Early dimensional change detected with high sensitivity	Smith & Knight identified limited early progression clinically	Digital monitoring enhanced early detection relative to conventional visual assessment

All studies evaluated IOS-based assessment of tooth wear and compared outcomes with conventional clinical indices, including BEWE (*n* = 3) [28, 31, 32], a study-specific modified Basic Erosive Wear Examination (mBEWE; *n* = 1) [27], and the Smith & Knight index (*n* = 1) [33].

Studies assessed anterior, occlusal, and full-dentition surfaces. Outcome measures were heterogeneous and included linear surface loss (µm), volumetric loss (mm³), diagnostic accuracy metrics (sensitivity/specificity), correlation coefficients, and receiver operating characteristic (ROC) analyses. Most investigations focused on enamel, with limited substrate-specific reporting.

### Quantitative detection of tooth wear by IOS

Across all included longitudinal studies, IOS-based methods detected and quantified surface change over time. Reported dimensional surface loss ranged from approximately 10 μm to over 500 μm, varying according to tooth type, surface, and duration of follow-up.

Over shorter follow-up intervals (approximately 12 months), quantified wear generally ranged between 10 and 150 μm [27]. In contrast, longer observation periods demonstrated substantially greater cumulative loss, exceeding 300–400 μm at 25 years [28], while 10-year follow-up showed a mean loss of approximately 137 μm [31].

Thresholds used to define meaningful progression differed between studies. One study defined clinically significant change as > 30 μm [31], whereas another demonstrated reliable detection of dimensional change at thresholds ≥ 200 μm [33]. Volumetric IOS analysis demonstrated moderate discrimination between surfaces classified as progression versus non-progression by BEWE (area under the receiver operating characteristic curve [AUC] = 0.80), with a − 1.22 mm³ threshold yielding 79% sensitivity and 61% specificity [32].

Collectively, these findings demonstrate that IOS-based quantification can detect relatively small longitudinal surface changes; however, the definition and interpretation of clinically meaningful ETW progression remain poorly standardised across the current literature.

### Comparison with conventional clinical indices

Conventional clinical indices demonstrated limited sensitivity to early longitudinal surface change and variable responsiveness across monitoring intervals. Over shorter follow-up intervals (≤ 12 months), categorical indices often showed no statistically significant change despite measurable surface change detected by IOS. In one study [27], mBEWE scores did not change significantly over approximately 12 months, whereas IOS detected measurable surface loss over the same period. Similarly, Smith & Knight identified limited change at the shorter follow-up interval, with greater progression observed by 12 months [33].

Agreement between IOS-derived measurements and BEWE scores was inconsistent, with correlations generally weak to moderate [28, 31]. This likely reflects the fundamental methodological difference between continuous quantitative outputs and ordinal categorical scoring systems, where categorical score progression occurs only after predefined thresholds are exceeded.

In longer-term follow-up studies (10–25 years), progression was demonstrated using both IOS and conventional indices [28, 31]. However, the ordinal structure of BEWE inherently limited sensitivity to incremental dimensional change compared with quantitative IOS measurements. Consistent with this, ROC analysis indicated that IOS-derived volumetric loss demonstrated moderate discriminatory performance for identifying BEWE-defined progression (AUC = 0.80) [32].

Overall, IOS-based quantification demonstrates greater sensitivity to early or short-term dimensional surface change, whereas conventional clinical indices remain more closely aligned with structured categorical severity assessment and longitudinal clinical risk stratification.

### Substrate and surface specific findings

Only limited stratification of outcomes according to substrate was reported across the included studies. Most investigations focused primarily on enamel surfaces, and only one study explicitly analysed both enamel and dentine [33]. Consequently, evidence regarding substrate-specific detection performance remains limited.

Surface-specific differences were observed across studies. Greater wear was most commonly reported on palatal anterior and occlusal molar surfaces compared with other anatomical regions [28, 31–33]. However, substantial heterogeneity in surface selection, outcome reporting, and analytical methodology limited direct cross-study comparison.

The absence of consistent substrate stratification limits interpretation of potential enamel- and dentine-specific differences in longitudinal monitoring performance and represents an important methodological gap within the current literature.

### Reproducibility and examiner agreement

Where reported, IOS-based measurements generally demonstrated acceptable reproducibility; however, formal measurement precision metrics (e.g., duplicate scans, intra-class correlation coefficients, or minimal detectable change) were not consistently reported. Conventional clinical indices demonstrated moderate to high inter-examiner agreement (κ = 0.65–0.87) [27, 31].

Calibration and blinding procedures were inconsistently reported. Variability in IOS superimposition workflows, alignment protocols, and progression threshold definitions further contributed to heterogeneity in longitudinal monitoring outcomes and interpretation.

### Methodological quality

Study quality was appraised using the Newcastle-Ottawa Scale (Online Resource 1). However, consistent with the aims of this review, emphasis was placed on conceptual and methodological heterogeneity rather than comparative quality scores [27, 28, 31–33].

### Synthesis of evidence

Given the marked variability in study design, follow-up duration, IOS systems, outcome metrics, and progression threshold definitions, quantitative meta-analysis was not undertaken.

Overall, the available longitudinal evidence indicates that IOS-based quantitative methods detect measurable surface change over time and demonstrate greater sensitivity to early longitudinal surface change than conventional clinical indices, particularly over shorter monitoring intervals. However, direct validation of IOS-derived measurements against high-resolution surface reference techniques, such as non-contact laser profilometry [34] was not reported in any of the included studies. This represents a critical limitation within the current evidence base and further complicates interpretation of quantitatively detected progression.

## Discussion

This review examined how longitudinal erosive tooth wear (ETW) progression is defined, measured, and interpreted across quantitative digital and conventional clinical monitoring approaches. The available evidence demonstrates that IOS-based methodologies consistently detect measurable dimensional surface change over time, including during relatively short monitoring intervals in which conventional categorical indices frequently demonstrate limited or no apparent progression. However, the findings also highlight a broader conceptual challenge within the current literature: quantitative digital measurements and conventional clinical indices assess fundamentally different dimensions of tooth wear progression despite frequently being interpreted as directly comparable indicators of progression. Consequently, observed differences in progression detection may reflect variation in measurement construct as much as differences in analytical sensitivity.

A central issue emerging from the evidence is that technological advancement in ETW monitoring has progressed more rapidly than the development of clinically meaningful interpretive frameworks. IOS systems are increasingly capable of detecting micron-level surface change; however, the clinical significance of these measurements remains poorly defined. At present, there is limited consensus regarding what magnitude of quantified surface loss represents physiological wear, early pathological progression, or clinically actionable disease activity. Consequently, greater analytical sensitivity does not necessarily translate into improved clinical understanding or decision-making. Without validated thresholds linking quantitative surface change to biological relevance, disease activity, and treatment need, interpretation of IOS-derived progression remains inherently uncertain.

### Clinical implications

From a clinical perspective, these findings suggest that IOS-based monitoring may provide additional value in situations where early change detection is a clinical priority, particularly in patients identified as high risk or undergoing preventive intervention. Conventional indices are designed for structured chairside assessment and reproducible risk categorisation [4–6]. However, by design, categorical scoring systems have limited resolution for detecting small incremental changes in surface loss [23]. IOS, by contrast, generates continuous quantitative data, allowing dimensional surface change to be quantified at substantially greater resolution [27, 28, 31–33].

The available evidence indicates that this increased analytical resolution permits detection of measurable surface change before categorical progression becomes apparent within conventional index systems. Clinical indices such as BEWE remain valuable for screening, severity categorisation, and risk assessment, but were not designed to quantify small longitudinal changes between clinical appointments. Quantitative IOS monitoring may therefore complement, rather than replace, conventional clinical assessment by providing a continuous measure of surface change over time.

Alignment between IOS-derived change measures and conventional indices varied across studies. Where correlations were reported, they were generally moderate to weak, even when statistically significant, reflecting the fundamental differences between continuous surface quantification and categorical clinical scoring. In one study, receiver operating characteristic analysis demonstrated that IOS-derived volumetric loss showed moderate discriminatory performance for identifying surfaces classified as progression versus non-progression by BEWE (AUC = 0.80) [32]. These findings support IOS-based quantification as an adjunctive tool rather than a direct replacement for index-based classification. Importantly, because BEWE served as the reference standard in these comparisons, quantitative measurements were not independently validated against a high-resolution reference technique.

One included study employed a study-specific modification of BEWE (mBEWE) [27] to facilitate longitudinal comparison of serial digital models. However, this approach should not be confused with the recently introduced BEWE 2.0 system [18], which was developed to address recognised limitations of the original index, including ceiling effects and limited discrimination across severity categories.

A further clinically important finding was the substantial variation in thresholds used to define meaningful tooth wear progression. Across studies, thresholds ranged from relatively small changes (e.g., > 30 μm) to substantially larger values (e.g., ≥ 200 μm), while some investigations did not define a progression threshold at all. This heterogeneity not only limits cross-study comparability, but also exposes a fundamental interpretive challenge within quantitative ETW monitoring. It is essential to distinguish between what is statistically detectable, what exceeds measurement variability, and what represents a change of genuine clinical relevance [35]. This challenge is not unique to quantitative monitoring. Although existing clinical index systems incorporate treatment recommendations based on categorical severity, these thresholds are largely consensus-based and have not been directly validated against quantified longitudinal surface loss. Consequently, uncertainty remains regarding how either categorical or quantitative measures should be translated into biologically meaningful and clinically actionable thresholds for preventive management, disease stabilisation, and restorative intervention.

Methodological heterogeneity was considerable and prevented quantitative meta-analysis. Studies differed in IOS systems, acquisition protocols, superimposition approaches, follow-up duration, surfaces analysed, and outcome metrics. In digital wear monitoring, superimposition methodology is a particularly important determinant of measured change [22, 25, 26]. Yet, the description and standardisation of superimposition strategies were variable, and stable reference regions were not consistently specified. Differences in trimming, inclusion/exclusion of gingival tissues, handling of restorations, and the choice of reference surfaces may all influence measured wear magnitude. Outcome reporting was also variable. Some studies expressed change as linear surface loss (µm), whereas others analysed volumetric loss (mm³), and statistical outputs were not consistently aligned. Collectively, these methodological inconsistencies limit cross-study interpretability and continue to impede development of validated, clinically meaningful frameworks for longitudinal ETW monitoring.

Evidence regarding substrate-specific monitoring performance remains limited. Studies primarily assessed enamel, and only one explicitly included both enamel and dentine. Given the differences in optical properties, surface morphology, and progression patterns between enamel and dentine [13], IOS detection performance and measurement precision are likely influenced by substrate composition. However, the existing longitudinal evidence does not allow firm conclusions regarding whether IOS-based monitoring performs more reliably on enamel or dentine.

Greater wear was reported on palatal anterior and occlusal molar surfaces, although methodological differences limited direct comparison. Consequently, it remains difficult to determine whether observed differences reflect true biological variation in wear patterns or methodological variation between studies. Future studies require more consistent surface-level classification and reporting of enamel and dentine involvement to improve interpretability and cross-study comparability.

Current evidence supports IOS as an adjunct to established clinical indices, which remain valuable for screening and provisional risk assessment. This is particularly relevant given that index-based assessments are embedded within current clinical workflows and guidance [16, 20], whereas IOS-based monitoring requires access to scanning technology, validated digital workflows, and an understanding of measurement thresholds [26, 29]. A pragmatic approach, supported by the current evidence, is to use clinical indices for initial screening and risk stratification, while selectively deploying IOS-based monitoring when precise longitudinal quantification is clinically justified, such as in high-risk patients, evaluation of preventive interventions and patient communication.

### Strengths and limitations

This systematic review employed a critical qualitative synthesis and was reported in accordance with PRISMA 2020 guidance to support methodological transparency in study identification, screening, and data extraction. Study selection and data extraction were performed independently by calibrated reviewers. However, the available evidence base remains limited, with only five longitudinal studies meeting the inclusion criteria. Although the included studies were generally methodologically acceptable, substantial heterogeneity existed across progression definitions, digital workflows, outcome measures, and interpretive thresholds, limiting direct cross-study comparison and preventing meaningful quantitative synthesis. Restriction to English-language publications may have introduced language bias. Formal assessment of publication bias was not undertaken due to the small number of included studies and substantial heterogeneity in methodology, outcome reporting, and progression definitions.

### Implications for future research

Strengthening the evidence base for longitudinal erosive tooth wear (ETW) monitoring will require greater methodological consistency and clearer conceptual frameworks for defining progression. In particular, future studies should clearly characterise the underlying tooth wear aetiology and report IOS systems and digital workflows transparently, including scanner model, software version, calibration procedures, operator training, and acquisition protocols. Superimposition workflows should also be described in sufficient detail to permit reproducibility, including reference region selection, management of artefacts, soft tissues and restorations, and any exclusions applied during analysis.

Standardisation of outcome reporting is also required. Quantitative outcomes should be reported using clearly defined and standardised metrics, including linear surface loss (µm) and/or volumetric change (mm³), together with repeatability, reproducibility, measurement precision, and minimal detectable change. Importantly, future research should move beyond demonstrating detectability alone and prioritise development of validated progression thresholds capable of distinguishing measurable dimensional change from clinically meaningful ETW progression. Achieving this will require integration of quantitative surface measurements with biological relevance, disease activity, functional impact, and clinically meaningful treatment thresholds.

Consistent surface-level reporting and explicit differentiation between enamel and dentine are required to improve cross-study comparability and clarify potential substrate-specific differences in monitoring performance. Addressing these methodological and interpretive challenges will be essential for developing clinically meaningful frameworks capable of integrating quantitative digital monitoring into routine longitudinal ETW assessment.

Future work should also consider development of a core outcome set (COS) for longitudinal ETW research. Informed by COMET guidance and the COS-STAD minimum standards [36, 37], this would provide a structured approach to defining the scope, involving relevant stakeholders, and reaching consensus on the minimum outcomes that should be measured and reported. Candidate outcome domains could include quantitative surface loss, categorical clinical progression, patient-reported symptoms or functional impact, and clinically relevant treatment consequences. Once these outcomes are agreed, further work would be required to determine how they should be measured, including appropriate digital workflows, measurement precision, and thresholds for clinically meaningful change.

## Conclusions

Current approaches to longitudinal ETW monitoring assess different dimensions of tooth wear and cannot presently be interpreted as directly interchangeable. IOS-based methodologies provide high-resolution quantification of dimensional surface change, whereas conventional clinical indices primarily support categorical clinical assessment and risk stratification. Although quantitative digital approaches demonstrate increased capability for detecting measurable early surface change, the clinical interpretation of these measurements remains uncertain due to the absence of validated frameworks defining meaningful progression thresholds. Advances in quantitative measurement have therefore outpaced current understanding of how dimensional surface change should be interpreted in relation to biological relevance, disease activity, and treatment need. Future research should prioritise development of standardised interpretive frameworks linking quantitative wear measurements to clinically meaningful ETW progression.

## Supplementary Information

Below is the link to the electronic supplementary material.

Supplementary Material 1 (DOCX 290 KB)

Supplementary Material 2 (DOCX 46.2 KB)

## Data Availability

All data supporting the findings of this review are contained within the article and its Online Resource.
