# Supplementary material for: Defining progression in erosive tooth wear: an interpretive challenge
Source: Clin Oral Investig. 2026 Oct 13;30(11):523. doi: 10.1007/s00784-026-07217-8 (PMC13661733; doi:10.1007/s00784-026-07217-8)
Supplement: Supplementary file 2 [file 784_2026_7217_MOESM2_ESM.docx]

## **Supplementary Table S1**. Full electronic search strategies

| Databases | Search strategy |
| --- | --- |
| MEDLINE-OVID  (n = 239) | #1 ((tooth wear) OR (erosive tooth wear) OR (tooth surface loss) OR (dental wear) OR (tooth erosion) OR (dental erosion) OR (tooth attrition) OR (tooth abrasion) OR (tooth demineralisation) OR (dental demineralisation) OR (tooth tissue loss) OR (TSL) OR (ETW) OR (BEWE) OR (Smith and Knight) OR (tooth wear index) OR (TWI) OR (clinical index) OR (dental model*) OR (study cast*) OR (dental impression*) OR (conventional impression*) OR (dental photography) OR exp Tooth Wear/ OR exp Tooth Erosion/ OR exp Tooth Demineralization/ OR exp Tooth Attrition/ OR exp Tooth Abrasion/)  AND  #2 ((intraoral scan*) OR (intraoral scanner*) OR (digital scan*) OR (three-dimensional imaging) OR (3D scan*) OR (IOS) OR (dental software) OR (digital assessment*) OR (digital monitoring) OR (virtual assessment) OR exp Dental Technology/ OR exp Software/ OR exp Imaging, Three-Dimensional/)  AND  #3 ((Basic Erosive Wear Examination) OR (BEWE) OR (Smith and Knight) OR (tooth wear index) OR (clinical index) OR (dental model*) OR (study cast*) OR (dental impression*) OR (silicone impression*) OR (alginate impression*) OR (conventional impression*) OR exp Dental Models/ OR exp Dental Impression Materials/ OR exp Dental Photography/) |
| Embase  (n = 626) | #1  ((tooth wear) OR (erosive tooth wear) OR (tooth surface loss) OR (dental surface loss) OR (dental wear) OR (tooth erosion) OR (dental erosion) OR (tooth attrition) OR (dental attrition) OR (tooth abrasion) OR (dental abrasion) OR (tooth demineralisation) OR (dental demineralisation) OR (tooth tissue loss) OR (abfraction) OR (enamel erosion) OR (dentin erosion) OR exp tooth wear/ OR exp tooth erosion/ OR exp tooth demineralization/ OR exp dental attrition/ OR exp dental abrasion/)  AND  #2 ((intraoral scan*) OR (intraoral scanner*) OR (digital scan*) OR (dental scan*) OR (three-dimensional imaging) OR (3D imaging) OR (IOS) OR (dental software) OR (digital assessment) OR (digital monitoring) OR exp intraoral scanner/ OR exp dental technology/ OR exp software/ OR exp dental software/)  AND  #3 (Basic Erosive Wear Examination) OR (BEWE) OR (Smith and Knight) OR (tooth wear index) OR (TWI) OR (clinical index) OR (dental model*) OR (study cast*) OR (study model*) OR (dental impression*) OR (silicone impression*) OR (alginate impression*) OR (polyvinyl siloxane impression*) OR (conventional impression*) OR (dental photography) OR exp dental model/ OR exp dental impression/ OR exp impression material/ OR exp medical photography/) |
| Web Of Science  (n = 28) | #1 TS=((tooth wear) OR (erosive tooth wear) OR (tooth surface loss) OR (dental wear) OR (tooth erosion) OR (dental erosion) OR (tooth attrition) OR (tooth abrasion) OR (tooth demineralisation) OR (dental demineralisation) OR (tooth tissue loss) OR (abfraction) OR (enamel erosion) OR (dentin erosion))  AND  #2 TS=((intraoral scan*) OR (intraoral scanner*) OR (digital scan*) OR (dental scan*) OR (three-dimensional imaging) OR (3D imaging) OR (IOS) OR (dental software) OR (digital assessment) OR (digital monitoring))  AND  #3 TS=((Basic Erosive Wear Examination) OR (BEWE) OR (Smith and Knight) OR (tooth wear index) OR (TWI) OR (clinical index) OR (dental model*) OR (study cast*) OR (dental impression*) OR (silicone impression*) OR (alginate impression*) OR (polyvinyl siloxane impression*) OR (conventional impression*) OR (dental photography)) |
| Scopus  (n = 132) | #1 TITLE-ABS-KEY((tooth wear) OR (erosive tooth wear) OR (tooth surface loss) OR (dental wear) OR (tooth erosion) OR (dental erosion) OR (tooth attrition) OR (tooth abrasion) OR (tooth demineralisation) OR (dental demineralisation) OR (tooth tissue loss) OR (abfraction) OR (enamel erosion) OR (dentin erosion))  AND  #2 TITLE-ABS-KEY((intraoral scan*) OR (intraoral scanner*) OR (digital scan*) OR (dental scan*) OR (three-dimensional imaging) OR (3D imaging) OR (IOS) OR (dental software) OR (digital assessment) OR (digital monitoring))  AND  #3 TITLE-ABS-KEY((Basic Erosive Wear Examination) OR (BEWE) OR (Smith and Knight) OR (tooth wear index) OR (TWI) OR (clinical index) OR (dental model*) OR (study cast*) OR (dental impression*) OR (silicone impression*) OR (alginate impression*) OR (polyvinyl siloxane impression*) OR (conventional impression*) OR (dental photography)) |

**Supplementary Table S2**. Detailed characteristics of included longitudinal erosive tooth wear monitoring studies

| **Study (Author, year)** | **Country** | **Design** | **Population** | **Age (mean ± SD)** | **Sample size** | **Follow-up** | **Conventional method** | **Digital workflow** | **Dentition / surfaces** |
| --- | --- | --- | --- | --- | --- | --- | --- | --- | --- |
| Bors et al., 2025 [31] | Romania | Prospective cohort | Adult | 24.7 ± 3.1 years | 517 | 10 years | BEWE | MEDIT i700; Exocad DentalCAD (Galway 3.1), best-fit registration | Maxillary anterior teeth: palatal and buccal surfaces. |
| Ellingsgaard et al., 2025 [27] | Denmark | Retrospective cohort | Paediatric | 11 to 16 years | 24 | 11-17 months (median 12 months) | mBEWE | TRIOS 3; 3Shape TRIOS Patient Monitoring v21.1, serial-model alignment | Central incisors, canines and first molars. |
| Marro et al., 2020 [32] | Belgium | Retrospective cohort | Paediatric | 12 to 13 years | 32 | 24-35 months | BEWE | TRIOS-digitised casts; WearCompare, selective surface alignment | Molar teeth: occlusal surfaces. |
| Maluf et al., 2025 [28] | Brazil | Retrospective cohort | Adolescent/young adult | 18.6 ± 1.11 years to 43.6 ± 1.11 years | 33 | 25 years | BEWE | CEREC Omnicam-digitised serial casts; Geomagic Control (3D Systems), tooth-level superimposition | Full dentition: occlusal surfaces. |
| García et al., 2022 [33] | Spain | Prospective cohort | Adult | 20 to 34 years | 46 | 1 year | Smith & Knight | 3M True Definition; Geomatic™ software (3D Systems), serial scan overlay/alignment | Full dentition: buccal/vestibular, lingual/palatal, and incisal/occlusal surfaces |

| **Study** | | **Measurement substrate / model type** | | **Aetiology** | | **IOS detection** | **Conventional detection** | **Reported conclusions** | |
| --- | --- | --- | --- | --- | --- | --- | --- | --- | --- |
| Bors et al., 2025 [31] | Digitised baseline casts and follow-up intraoral scans (enamel surfaces) | | Natural Tooth Wear | | Baseline vs follow-up (10 years) tooth wear measurement - 137 ± 79 µm (mean loss).  Palatal surfaces were lost to a greater extent (162 ± 85 µm) compared with buccal surfaces (114 ± 66 µm), p = 0.002.  Clinically significant threshold defined >30 µm surface loss. | | 1. Baseline BEWE - score 0.  2. Follow-up BEWE -   - Score 0: 300 participants - Score 1: 120 participants - Score 2: 70 participants - Score 3: 27 participants   High inter-examiner agreement: κ 0.87 (p < 0.001).  Moderate correlation with IOS: r = 0.58 (p < 0.001). | | IOS-based quantification demonstrated greater sensitivity to incremental enamel surface change than BEWE. However, both approaches provided complementary information, with BEWE remaining useful for clinical screening and categorical severity assessment. |
| Ellingsgaard et al., 2025 [27] | In vivo enamel surfaces (digital models) | | Natural Tooth Wear | | Quantitative tooth wear assessment baseline vs follow-up (~12 months).   - Incisors: median 80 (30-310) µm; mean 100 (SD 60) µm; n=92 - Canines: median 110 (0-560) µm; mean 150 (SD 120) µm; n=88 - Molars: median 90 (0-400) µm; mean 120 (SD 80) µm; n=94 - Overall: median 90 (0-560) µm; mean 120 (SD 90) µm; n=274   Wear differed by tooth type (p = 0.007), with canines > incisors (p = 0.005), and no significant differences for incisors vs molars (p = 0.661) or canines vs molars (p = 0.162). | | mBEWE at baseline and follow-up showed no significant difference (p = 0.132).  Significantly higher mBEWE scores were observed in anterior sextants compared with posterior teeth (p < 0.05).  Moderate inter-examiner agreement: κ = 0.652 (p < 0.001). | | mBEWE showed no significant change in tooth wear progression over a ~12-month period. In contrast, IOS-based quantitative analysis identified significant, tooth type dependent wear. These findings indicate that IOS offers greater sensitivity for short-term monitoring of erosive tooth wear. |
| Marro et al., 2020 [32] | Digitised casts (enamel surfaces) | | Natural Tooth Wear | | Occlusal volumetric tooth wear quantified from IOS-digitised casts (96 first permanent molar surface pairs).  Surfaces with BEWE defined progression (n = 54) showed significantly greater volume loss than non-progression surfaces (median −2.19 vs −0.37 mm³; p < 0.001).  ROC analysis demonstrated good discrimination between progression and non-progression surfaces (AUC = 0.80); a −1.22 mm³ threshold yielded 79% sensitivity and 61% specificity. | | Visual assessment of BEWE progression on occlusal first molars (54 progression vs 42 no progression surfaces). | | Quantitative volumetric analysis of IOS digitised cast scans differentiated surfaces with visually detected BEWE progression from those without. Diagnostic performance was moderate, supporting IOS-based quantification as an adjunct to clinical assessment rather than a stand-alone diagnostic tool. |
| Maluf et al., 2025 [28] | Digitised serial casts (enamel surfaces) | | Natural Tooth Wear | | Quantitative 3D tooth wear by group (baseline → 25 years):  Maxillary   - Anteriors: 332 μm - premolars: 282 μm - First molars: 475 μm - Second molars: 494 μm   Mandibular   - Anteriors: 332 μm - Premolars: 279 μm - First molars: 398 μm - Second molars: 464 μm | | BEWE by sextant (baseline → 3 years → 25 years):  Maxillary   - Right posterior: 0.58 → 0.91 → 1.33 - Anterior: 0.34 → 0.41 → 0.60 - Left posterior: 0.42 → 0.83 → 1.23   Mandibular   - Left posterior: 0.64 → 1.21 → 2.03 - Anterior: 0.22 → 0.33 → 0.51 - Right posterior: 0.71 → 1.11 → 2.09 | | Quantitative analysis demonstrated greater sensitivity to incremental tooth wear progression than BEWE, while BEWE remained useful for clinical screening. Digital monitoring supported longitudinal assessment; however, correlations between BEWE and 3D measurements were statistically significant but weak. |
| García et al., 2022 [33] | Enamel and dentine surfaces (in vivo) | | Natural Tooth Wear | | IOS compared with Smith & Knight:  Overall performance: Sensitivity 100%, Specificity 84.9%, PPV 71.0%, NPV 100%, Accuracy 89.0%.  Surface specific performance:   - Palatal/lingual surfaces - Sensitivity 91.2%, Specificity 58.6%, PPV 100%, NPV 92.2% - Occlusal surfaces - Sensitivity 100%, Specificity 83.5%, PPV 59.8%, NPV 100%   IOS reliably detected dimensional changes ≥200 μm. Demonstrated greater sensitivity to early tooth wear progression than Smith & Knight. | | Smith & Knight with % wear change:  Control 1 (6 months)   - Antero-superior: 5/46 (9.3%) - Postero-superior: 3/46 (7.0%) - Antero-inferior: 3/46 (7.0%) - Postero-inferior: 3/46 (7.0%)   Control 2 (12 months)   - Antero-superior: 21/46 (46.0%) - Postero-superior: 34/46 (73.0%) - Antero-inferior: 21/46 (46.0%) - Postero-inferior: 25/46 (54.1%)   Smith & Knight detected relatively little progression at 6 months (7-9%), but substantially more by 12 months, especially postero-superior (~73%). | | IOS demonstrated greater sensitivity for early change detection than visual Smith & Knight assessment. IOS supports use as a diagnostic adjunct to conventional indices, particularly for early detection and patient monitoring. |

| **Supplementary Table S3**. The Newcastle-Ottawa Scale (NOS) for assessing the quality of non-randomized studies | | | | | | | | | |
| --- | --- | --- | --- | --- | --- | --- | --- | --- | --- |
| **Reference** | **Selection** | | | | **Comparability** | **Outcome** | | | **Overall quality assessment NOS score (0-9)** |
|  | **Representativeness of the exposed cohort** | **Selection of the non-exposed cohort** | **Ascertainment of exposure** | **Demonstration that outcome of interest was not present at start of study** |  | **Assessment of outcome** | **Was follow-up long enough for outcomes to occur?** | **Adequacy of follow up of cohorts** |  |
| Bors 2025 et al., [31] | Somewhat  representative | Drawn from the same community | Secure record | Yes | Study controls for tooth-wear aetiology. | Record linkage | Yes (≥12 months) | Loss to follow-up unlikely to introduce bias | 8 |
| Ellingsgaard et al., 2025 [27] | Somewhat  representative | Drawn from the same community | Secure record | Yes | Study controls for tooth-wear aetiology. | Record linkage | Yes (≥12 months) | Complete* | 9 |
| Marro 2020 et al., [32] | Selected group  of users | Drawn from the same community | Secure record | Yes | Study controls for tooth-wear aetiology. | Record linkage | Yes (≥12 months) | Loss to follow-up unlikely to introduce bias | 7 |
| Maluf 2025 et al., [28] | Selected group  of users | Drawn from the same community | Secure record | Yes | Study controls for tooth-wear aetiology. | Record linkage | Yes (≥12 months) | Loss to follow-up unlikely to introduce bias | 7 |
| García 2022 et al., [33] | Selected group  of users | Drawn from the same community. | Secure record | Yes | Study controls for tooth-wear aetiology. | Record linkage | Yes (≥12 months) | Complete* | 7 |

* No samples or subjects were reported as lost, missing, or excluded without explanation.
